# Effects of Auditory Training on Speech Recognition in Children with Single-Sided Deafness and Cochlea Implants Using a Direct Streaming Device: A Pilot Study

**DOI:** 10.3390/jpm13121688

**Published:** 2023-12-05

**Authors:** Stefanie Muck, Astrid Magele, Bianca Wirthner, Philipp Schoerg, Georg Mathias Sprinzl

**Affiliations:** 1 Department of Otorhinolaryngology, Head & Neck Surgery, University Clinic St. Poelten, 3100 St. Poelten, Austria; stefanie-muck@aon.at (S.M.); astrid.magele@stpoelten.lknoe.at (A.M.); bianca.wirthner@stpoelten.lknoe.at (B.W.); philipp.schoerg@stpoelten.lknoe.at (P.S.); 2 Karl Landsteiner Institute of Implantable Hearing Devices, 3100 St. Poelten, Austria

**Keywords:** SSD-CI children, rehabilitation training, direct auditory input

## Abstract

Treating individuals with single-sided deafness (SSD) with a cochlear implant (CI) offers significant benefits for speech perception in complex spatial listening environments. After implantation, training without involvement of the normal-hearing ear is essential. Therefore, the AudioLink streaming device (MED-EL GmbH, Austria) can be used to connect the externally worn audio processor to media devices; thus, the auditory stimuli are directly streamed to the implanted ear. The aim was to test whether children with SSD, aged 5–12 years, accept this training method and whether auditory training, streamed directly via AudioLink using the Tiptoi device (Ravensburger GmbH., Ravensburg, Germany), improves speech recognition. A total of 12 children with SSD and implanted with a CI received Tiptoi training via AudioLink and were asked to practice daily for 10 min over a period of one month. All participants completed the training. The measurements employed to assess improvement included speech audiometry, speech, spatial, and quality of hearing scale for parents (SSQ P), and specially designed tasks crafted for this study. Daily training of 9.93 min was reported. The word recognition score (WRS) at 65 dB and 80 dB in aided condition significantly improved and the WRS streamed via AudioLink was significantly better after training. The speech, spatial, and qualities of hearing scale for parents (SSQ P questionnaire) showed significant improvement in the dimension of quality of hearing and overall gain. The outcomes of the Tiptoi tasks resulted in a significant benefit in both categories of the “recognition of sounds” and “understanding of sentences”. The results are very encouraging and do not only show the positive uptake of daily training at home but also how this resulted in a significant improvement in subjective and objective measures for this rather short training period of one month only.

## 1. Introduction

Approximately 466 million people worldwide have disabling hearing loss, and 34 million of these are children [1]. Newborn screening detects one case of SSD per thousand births worldwide, and this number rises to three cases per hundred children globally by the time they enter school [2,3,4]. In Austria, 1–2 children in 1000 are born with hearing impairment and between 0.1 and 5% of them are affected by unilateral hearing loss, i.e., single-sided deafness (SSD) [1]. SSD is characterized by severe to profound sensorineural hearing loss in one ear (pure-tone average (PTA) 4 (average of 0.5, 1, 2 and 4 kHz) ≥ 70 dB) and normal hearing (PTA4 ≤ 30 dB) in the other ear [5]. One option for the rehabilitation of SSD is the implantation of a cochlear implant (CI). The first CI implantation in SSD was conducted in 2003 in Belgium [2,6]. Subsequently, CI implantations in SSD were consistently carried out as part of ongoing research [2,6,7]. In 2013, MED-EL implants obtained the CE mark, extending the indication for SSD and asymmetric hearing loss (AHL) to both adults and children. This marked a significant development, enabling clinicians in the European Union and countries recognizing the CE mark to officially perform CI implantations in both adults and children with SSD [2]. In 2019, MED-EL implants secured FDA approval for its CI device to be implanted in patients with AHL/SSD, thereby globally expanding the indication criteria for CI to include the treatment of SSD [2,8]. Before that, the rehabilitation of SSD was limited to transferring signals from the deaf ear to the contralateral ear. Therefore, only conventional hearing aids, such as CROS hearing aids or bone-conducted technologies, were used [9]. With the hearing implant indications constantly evolving, treatment of SSD with a CI is becoming a routine procedure in quite a few countries, such as Austria [10]. A number of studies demonstrated the benefits of unilateral cochlear implantation in adult individuals with SSD, including improvement in speech perception in noise, sound localization, subjective hearing performance, and improvements in daily life as well as combat against persistent tinnitus [6,7,11,12]. The use of a CI offers individuals with SSD significant benefits for sound localisation and speech perception in complex spatial listening environments and the improved ability to perceive competing voices in challenging listening situations [13,14,15,16,17]. Cochlear implantation in SSD improves the localization of sound sources in both children and adults [15,18,19]. The effects of SSD extend not only to spatial hearing, but also to language development. Associations of SSD with significantly poorer spoken language and slower educational progress were found [9]. Most important is that CI intervention for congenital deafness, no matter if bilateral or SSD, should be carried out as early as possible because outcomes are better when the period of auditory deprivation is shorter [9]. Despite very modern automatic sound management and noise filters, the healthy ear will always dominate at SSD. The challenge for individuals with SSD is the integration of natural hearing on one ear and electronic hearing on the other ear [20].

After implantation, the brain requires time and training to adjust to auditory signals from both sides, facilitating the natural perception of speech and environmental sounds. The aim is to provide binaural hearing by enhancing the perceptual integration between the implanted ear and the healthy ear [5]. Therefore, this new and different way of hearing (electronic hearing) needs to be practised without the normal-hearing ear, usually the prominent ear, being involved [12]. Extensive auditory training is crucial for achieving improvements in SSD-CI patients, irrespective of age, as indicated by various studies [5,11,12,21].

While there is a wealth of published resources on auditory training for the bilaterally deafened or hearing-impaired paediatric population, it is less frequently carried out for adult CI users or unilaterally deafened children. Additionally, in Austria, increasingly less clinics offer one-to-one auditory rehabilitation due to financial constraints and the insufficient number of therapists available for the rehabilitation of CI users. The rehabilitation training of children with SSD especially involves problems, such as regular, at-home, and daily training to ensure success. Practice material for auditory training with children is scarce. In case of SSD, the normal-hearing ear is only covered manually with an earplug or similar, during auditory training, which at home is often performed incorrectly [22]. Even in professional auditory training sessions at the clinic, there is always a certain risk that the subject listens with the normal-hearing ear during the exercises; hence, the implanted ear does not become trained properly. To circumvent this problem and ensure adequate stimulation during auditory training, it is recommended to deliver the stimulus directly to the implanted ear through direct auditory input (DAI) [12]. DAI is the direct transmission of audio signals to the audio processor. For at-home training, DAI is the only way to bypass the healthy ear and, thus, train the implanted ear. The newest generations of implants, such as the SYNCHRONY 2 cochlear implant, feature an external universal connectivity device, AudioLink (MED-EL GmbH, Innsbruck, Austria). This device can be used to connect the externally worn audio processor with a phone, tablet, TV, or other media device [23]. AudioLink allows for the direct transmission of the auditory stimuli via streaming to the implanted ear. In the present study, the use of AudioLink for rehabilitation training in SSD-CI children was tested for its acceptance and effectiveness.

During the COVID pandemic, access to hearing rehabilitation was delayed or more difficult for CI users. The partial lockdown situation has shown that the use of technical media (apps, computer programs, etc.) offers an additional therapy and rehabilitation approach in such situations, in particular, but also in the future in general [24]. The literature review showed that modern auditory training may increase the motivation in children, and that even a minimum of additional auditory training may significantly increase the success of the therapy [25,26]. The use of additional computer programs improves the success of the therapy in children with a CI. Gamification may increase the motivation of the children and thus accelerate and positively support the learning process. Using gamified computer programs should decrease dropout rates and enhance the efficacy of auditory training. The essential element is to make the process more engaging by utilizing computer programs, assisting users in improving their speech comprehension skills [25,27,28]. This clearly shows that the use of a suitable and age-appropriate digital toy in the therapy may increase motivation because it takes advantage of the gamification factor with its digital component.

In the study, we decided to connect the Tiptoi digital learning game to AudioLink for therapeutic purposes. This toy is used by many children in daily play. Playing a game is more accepted by kids than conventional exercises for training speech perception on the implanted ear. Practicing takes time but, by integrating it into daily play, practice is more likely to happen. By connecting Tiptoi to AudioLink, the implanted ear can be trained specifically while playing.

The aim of this pilot study was to ascertain whether children with SSD aged 5–12 years accept this training method and whether it improves speech recognition using Tiptoi training directly streamed via AudioLink.

## 2. Materials and Methods

Design: The study has a prospective within-subject repeated-measures design in which each subject serves as its own control. It is intended to compare the subject’s performance before and after auditory Tiptoi training through direct streaming via AudioLink. 

Participants: A total of 12 children with SSD aged 5–12 years who were routinely implanted with a CI received Tiptoi training via AudioLink as an additional voluntary therapy intervention and asked to practice with this tool 10 min per day for one month. In Austria, children do not receive rehabilitation at the implanting clinic as part of the standard implant program. There is no mandatory therapy program throughout the country that is carried out after implantation. Parents can organize therapy with local professionals. The study involved the 12 most recently implanted children in the clinic, who met the inclusion criteria. The inclusion criteria also involved children who had undergone CI implantation more than three months ago, ensuring a sufficient number of subjects for analysis. Potential participants were asked during routine examinations at the audiology department in the clinic if they wanted to participate in the study. Procedures: An informed consent form was signed by parents and children. The ethics approval was given by the Ethic Commission of Lower Austria with the EK number GS1-EK-3/198-2021.

A diary was developed to record the practice time per day and the training material used. Audiometric and subjective measures were carried out to compare the word and speech recognition performance before auditory training and one month after auditory training. In this diary, the daily training time and the training materials used were recorded. Additionally, a self-report scale was provided where the subject could indicate their level of understanding for the day. The scale ranges from zero to ten, representing the spectrum from no understanding to perfection.

Table 1 shows the tests performed before (baseline, BL) and after (F/U) the Tiptoi training. The subjects were evaluated at these two independent sessions and the results were compared. The tests were performed by an audiologist at the St. Pölten University Clinic.

### 2.1. Audiologic Tests

To identify the word recognition score (WRS), the Göttingen Children’s Language Test was used. This is a commonly used test procedure in German-speaking countries consisting of monosyllabic nouns that can typically be found in children’s vocabulary. The words are the repertoire of the Freiburg speech intelligibility test, that was created according to DIN 45621. In each test procedure, 10 items were presented through the loudspeakers in a sound field in the audiometry booth, and the children were asked to repeat the words [29,30,31]. The contralateral ear was masked with a narrowband noise of the same intensity as the speech signal (signal-to-noise ratio (SNR) 0). The test was performed in the sound field at 65 dB and 80 dB and streamed via AudioLink. For the streaming category, the AudioLink streaming device was connected to the audiometer. The AC440 hardware and the equinox suite software were used for this purpose.

Each subject was given 10 exercises directly streamed via AudioLink (DAI) to test if they can recognize sounds from the Tiptoi book and 10 exercises to test if they can understand sentences as a baseline (BL) measurement. To avoid bias, the tasks were carefully selected and compared to ensure the same semantic field and the same level of difficulty of the tasks before and after the intervention. For the post-training evaluation session (F/U) another semantically similar book with slightly different exercises was used to avoid a trough repetition set learning effect. 

The equipment handling, technical details, and training possibilities at home were explained in the course of a therapy unit. The connection of the devices was tested for at least 1 month.

The original idea was that the parents test and document a set of streaming exercises per week at home (10 sounds and 10 sentences), as it was tested at BL and F/U. For these weekly tasks, they were given a special test protocol with a description of how to instruct their child and how to document the results of the exercises.

### 2.2. Subjective Questionnaire

The Speech, Spatial, and Qualities of Hearing Scale for Parents (SSQ P) is a caregiver-reported patient-reported outcome and was designed to measure auditory disability across a wide variety of domains, reflecting the reality of hearing in everyday life. The 22 questions are categorized into three sections: speech (covering the perception of speech in diverse situations and listening settings), spatial hearing (addressing the perception of direction, distance, and localization of sounds), and qualities of hearing (encompassing the identification of sound, segregation of sound sources, clarity, and naturalness) [32,33,34]. The question involves a visual analog scale (VAS) ranging from 0 to 10. The totals for each section were computed as the mean of their item’s VAS scores (0–10). The overall score represents the mean of the three sections. According to several studies, this test is a reliable and valid evaluation material for hearing impaired children [32,33,35,36]. 

The SSQ P was administered twice—before and after the Tiptoi training to survey the subjective dimension and the daily challenges of the subjects. 

### 2.3. Equipment

AudioLink allows the connection of children’s favourite digital toys, tablets, or smartphones, and makes DAI possible. This way, the auditory training can be integrated into daily play. In our study, we connected the Tiptoi game to AudioLink for therapeutic purposes.

Tiptoi is a digital audio learning game consisting of a digital pen and a game board, book, or puzzle with digital paper. Children can use an electronic pen when interacting with Tiptoi books, games, puzzles, or even a globe. The pen has a built-in microphone. By being placed on various parts of the printed surface, the pen identifies the tapped area through an invisible dot grid. The infrared scanner in the pen reads this code and, in its simplest case, plays audio files explaining the game or the pictures, imparting knowledge, or vocalizing the content of a book. 

The game is suitable for the training purpose due to its playful and interactive character, relying on what the player has heard and comprehended.

**Figure 1:**
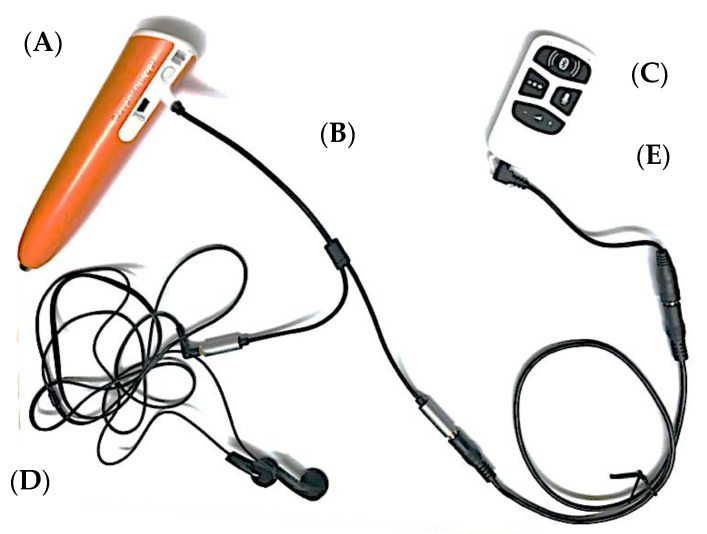
Cable connection: (A) Ravensburger Tiptoi pen; (B) UGREEN Y adapter audio splitter; (C) MED-EL AudioLink; (D) Panasonic in-ear headphones; (E) AudioLink cable accessories.

An audio splitter was required so that AudioLink and Tiptoi can be connected, and the examiner (therapist and parents at home) can also control the auditory inputs using commercially available headphones. Figure 1 shows the cable connection of Tiptoi, AudioLink, and headphones to the audio splitter.

The entire setup—AudioLink, cables, Tiptoi pen, Tiptoi books, audio splitter, headphones—was provided to the subjects free of charge for the entire study period.

### 2.4. Statistics

This was a prospective study. By using a within-subject design, the effect of variability was minimized such that treatment effects could be evaluated. The use of standardized evaluation methods assured the reliability of the data across investigational centers. For the statistical analysis and presentation of the results, GraphPad Prism was used. Heterogeneity and variability inherent to the population and their outcome measures were recognized.

Descriptive statistics were used to report patient demographics and baseline characteristics. Quantitative data were presented as mean, standard deviation (SD), and range (minimum and maximum); qualitative data were presented as absolute and relative frequencies.

Inferential statistics were performed to detect differences between before and after Tiptoi training via AudioLink. Depending on the distribution of the outcomes, either a parametric Student’s t-tests or nonparametric Wilcoxon signed rank tests were conducted, depending on the data distribution. The Kolmogorov–Smirnov test was used to check for data distribution. Comparison of the performance outcomes from the first and the second evaluation point was performed by calculating the difference between the mean values. Performance outcomes from the baseline condition compared with the F/U were calculated and tested for significance. 

## 3. Results

Each of the 12 included subjects completed the four-week training described.

### 3.1. Subject Demographics

Table 2 shows the subject demographics. The present study included six female and six male subjects. Seven patients were implanted on the right side and five were using a CI on the left side. The mean age at surgery was 6.1 years. The mean age at Tiptoi training was 8.88 years. Mean wearing time at BL was 9.42 h, and mean wearing time at F/U was 10.92 h. This is a significantly increased daily wearing time of 1.5 h mean (*p* = 0.0241). This means that the daily wearing time could be increased by an average of 15.92%. 

The reported average daily training time was 9.93 min. Five participants exercised for more than 10 min daily, while two subjects had an average training duration between 9 and 10 min per day. The remaining participants, as per their training diaries, engaged in training sessions lasting between 5 and 8 min. 

### 3.2. Word Recognition Score (WRS)

**Figure 2:**
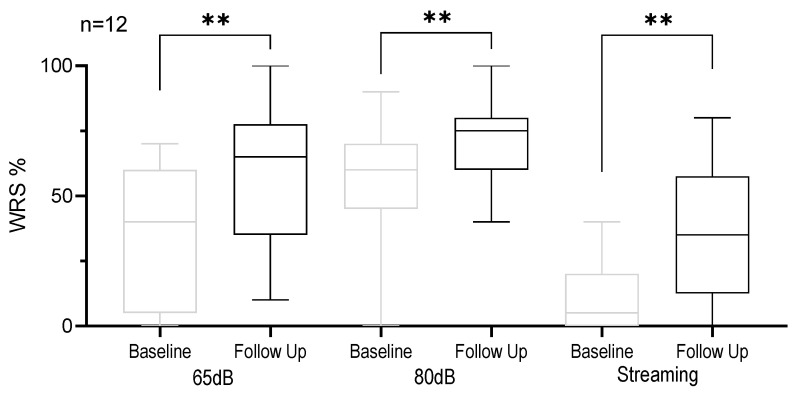
Boxplot analysis of word recognition score determined by Göttingen children speech test. The boxplot displays the distribution of word recognition scores at 65 dB, 80 dB, and streamed at baseline and follow up. Horizontal lines denote the median. The whiskers extend to the minimum and maximum values. ** *p* < 0.001.

The results of the performed studies showed a significant improvement in the WRS. The WRS at 65 dB and 80 dB in aided condition significantly improved (*p* = 0.0038 and *p* = 0.0055). This corresponds to an improvement of 57.2% at 65 dB and 15% at 80 dB. The WRS streamed via AudioLink ensuring a direct auditory input through the implant was 26.48% better after Tiptoi training (*p* = 0.0008). So, the speech recognition in quiet improved significantly. Figure 2 shows the results of the WRS.

### 3.3. Tiptoi Tasks

**Figure 3:**
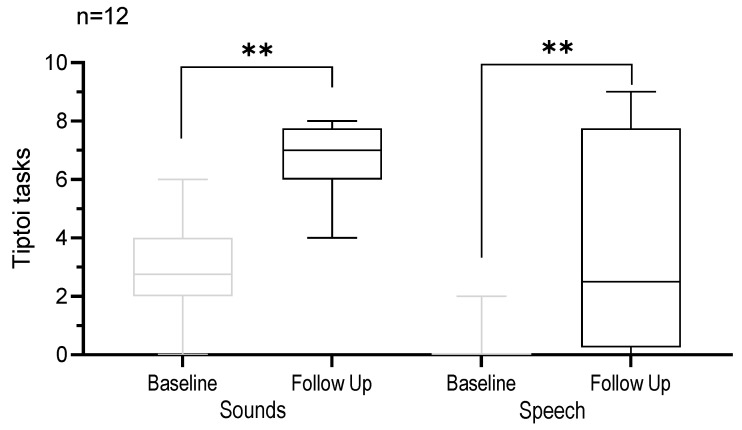
Boxplot analysis of Tiptoi tasks, sounds, and speech at baseline and follow up. Horizontal lines denote the median. The whiskers extend to the minimum and maximum values. ** *p* < 0.001.

Figure 3 shows the outcomes of the Tiptoi tasks. Significant benefits were observed in both the “recognition of sounds” and “understanding of sentences” categories (*p* = 0.0001 and *p* = 0.0027). Specifically, there was a 128.57% enhancement in sound recognition and a 1075% improvement in sentence understanding. 

### 3.4. SSQ P

**Figure 4:**
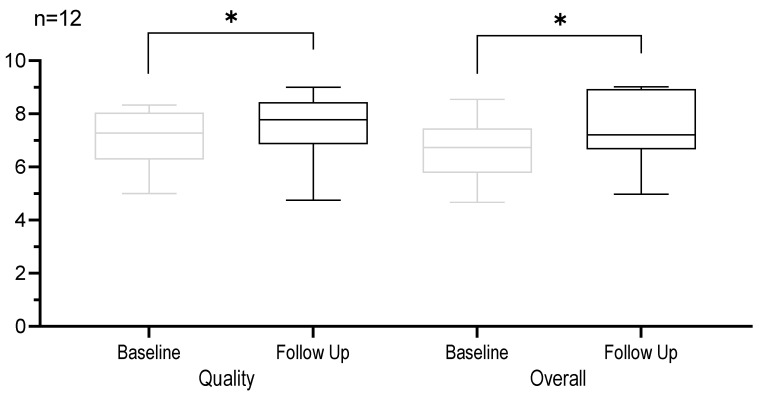
Boxplot analysis of the individual dimension quality of hearing and overall gain of speech, spatial, and quality of hearing scale for parents represented at baseline and follow up. Horizontal lines denote the median. The whiskers extend to the minimum and maximum values. * *p* < 0.05.

The SSQ P showed a significant improvement in the dimension of quality of hearing with a mean increase of 33.04% (*p* = 0.0497). The overall gain increased significantly by an average of 28.49% (*p* = 0.0243). The categories speech and spatial showed tendencies towards improvement after training but the results were not significant. Figure 4 shows the dimensions of the SSQ P questionnaire.

## 4. Discussion

The study showed that, as already demonstrated for SSD-CI adults, SSD-CI children may also benefit from training via direct streaming [12]. 

The primary aim of the study was to access the acceptability of this training method. This was evaluated by means of compliance and completion of the training. All 12 study subjects successfully completed the 4-week training without any dropouts, indicating a high level of acceptance by the target group for this method. The recommended training time of ten minutes per day was nearly fulfilled by an average training time of 9.93 ± 3.89 min. Although the training diary was carefully completed, the execution of the weekly tasks that the parents were supposed to do with the children was not successful. The self-report scale was partially completed only, rendering the data non-evaluative. Due to the missing compliance of the parents, the number of results was too small and the submitted protocols were incomplete and, thus, not evaluable. The increase in the WRS indicated that the subjects significantly improved their speech recognition in silence, i.e., without background noise, at the implanted ear in just one month of Tiptoi training. 

The reason for testing both conditions, WRS in a sound field and streamed, is susceptibility to error caused by informal masking [37]. This is often observed in children in clinical settings. Additionally, even with proper masking, in a sound field, the potential for sound to reach the healthy ear cannot be completely ruled out. Consequently, both conditions—sound field and streamed via AudioLink—were examined to establish a comprehensive set of measurements.

The variability in WRS can be attributed to the diverse backgrounds of the subjects. A few were recently implanted and just adapting to the CI; they tended to show the biggest improvement through training. The majority of the subjects have been CI users for a longer duration. They were adapted to the CI and without therapy; there is usually no substantial increase in performance. Therefore, it was possible to assess how the training itself affects performance. The statistical impact on the results with regard to the duration of treatment with the CI at the time of training is not evaluable due to the small number of participants.

The improvement at Tiptoi tasks signified that the subjects understood sounds and speech via DAI much better after the training. So, a training effect in understanding DAI can be observed here. The enhancements noted in Tiptoi tasks and the outcomes of the streamed WRS suggest that the improvements extend beyond sounds and speech within the Tiptoi book; it also can be transferred to activities like making phone calls or listening to streamed videos on mobile phones. This reasoning suggests that children could potentially benefit from the training in these diverse listening situations.

The results of the SSQ P indicated that the parents also noticed improvement in everyday life, which means subjective alterations were perceived. This is at least as important in rehabilitation as the objective results. For this reason, training via streaming should be included as a training method in the rehabilitation of SSD-CI children. This is particularly crucial as the use of DAI is the only way to effectively train the implanted ear without the healthy ear listening in, which minimizes the effectiveness of training. Regarding the results of the SSQ P questionnaire, the daily wearing time increased significantly. This was reported by parents, but in this case an increase in wearing time can be seen as a result of the benefits of the training in everyday life.

The problem is still that there is no specific, validated exercise material for the described purpose and target group. Tiptoi accessories were discovered to be successful, appealing, and suitable for this target group. Further research could explore connecting other digital toys for children receiving DAI to identify options that are well suited for the rehabilitation process in this target group.

These findings agreed with already existing research results showing that modern auditory training increases motivation through the gamification factor and that even short sequences of such auditory training measurably improve the success of the therapy [25,26,27,28]. If children’s toys are used in addition, the training can be integrated into everyday life and no extra time is required for the training. Therefore, it will be carried out more consequently and the outcome will be better.

The results of the study are consistent with the following research findings: The use of technical media can be used in addition to therapy. Using appropriate therapy material and a suitable, precise training program increases motivation in children, in particular [24]. It is important to note that at-home training or streaming training does not replace the clinical rehabilitation supervision and frequent audiological and technical checks. It is recommended to perform streaming training in addition to ensure practice in daily play.

Study limitations:

While the study contributes valuable insights to the understanding of the design of auditory training for SSD-CI children, it is important to acknowledge certain limitations. The sample size was relatively small, limiting the generalizability of the findings; therefore, the present work should be seen as a pilot study. The follow-up time in the study was comparatively short, and this may limit our ability to assess the long-term sustainability and enduring effects of the AudioLink training implemented. The study did not include a comparison with a control group, making it challenging to benchmark the effectiveness of the AudioLink training employed in our research. Another limitation is that the start of the AudioLink training varies among subjects in relation to the time since the implantation. This means that the subjects are not all at the same level, leading to varying conditions and, thus, the results cannot be generalized. 

The current study utilized a relatively small set of 10 items for scoring in speech audiometry, potentially limiting the depth of assessment of participants’ speech recognition abilities.

Future studies should aim for larger and more diverse samples to strengthen the external validity of the results. Further research should incorporate extended follow-up periods to provide a more comprehensive understanding of the lasting impact of the training intervention. Future studies should incorporate a control group including children without AudioLink training to assess its relative efficacy. Further research could standardize the starting point of the AudioLink training or carefully account for variability, allowing for more accurate comparisons and generalizability of results. Future studies could explore the inclusion of a more extensive set of speech recognition items. This could provide a more comprehensive understanding of participants’ speech perception capabilities and contribute to refining assessment methodologies.

Addressing these limitations in future research will contribute to a more robust understanding of the effectiveness and applicability of AudioLink training for SSD-CI children.

## 5. Conclusions

Our aim was to show the possibilities for SSD-CI training in children excluding the normal hearing ear while only training the implanted ear using the AudioLink streaming device. The results are very encouraging, not only reflecting a positive uptake but also implying that the training seems acceptable, as demonstrated by the consistent completion of daily home training. The training shows the potential to result in significant improvement in subjective and objective measures for this rather short training period of one month only. The key factor for successful rehabilitation training in children is motivation. It can be given by the application of appealing training material like toys. The connection of children’s favorite digital toys like Tiptoi with AudioLink may facilitate motivating training of the implanted ear.

## Figures and Tables

**Table 1:** Test procedure.

Test Procedure Baseline and Follow Up
**Audiologic tests**
Göttingen Children Speech Test @65dBHL
Göttingen Children Speech Test @80dBHL
Göttingen Children Speech Test Streaming via AudioLink
Tiptoi Exercises Via AudioLink ◦ 10 Sounds ◦ 10 Sentences
**Subjective questionnaire**
SSQ P- Speech, Spatial, and Quality of Hearing Questionnaire for Parents

**Table 2:** Subject demographics.

Patient ID	Implanted Ear	Gender	Age at Surgery (Years)	Age at Training (Years)	Daily Wearing Time BL (h)	Daily Wearing Time F/U (h)	Daily Training Time (Mean in min)
01	left	male	5.67	8.08	7	12	17.86
02	right	male	6	7.58	12	12	7.32
03	right	male	4.92	8.42	7.5	8	11.07
04	right	male	2.92	8.75	6.5	12.5	5.71
05	right	male	8.33	11.75	12	13	8.21
06	left	female	5.75	9.08	10	10	5.18
07	left	female	2.42	10.33	7	8.5	5.54
08	right	female	8	8.42	10	10	9.11
09	right	female	7.12	7.67	12	12	14.11
10	left	female	9	9.25	12	14	15.36
11	left	male	6.75	7.83	6	7	10.36
12	right	female	7.5	8.5	11	12	9.29
Mean			6.1	8.88	9.42	10.92	9.93
SD			2.11	1.17	2.44	2.31	3.89

## Data Availability

Data available upon request by the corresponding author.
